# Chyawanprash, An Ancient Indian Ayurvedic Medicinal Food, Regulates Immune Response in Zebrafish Model of Inflammation by Moderating Inflammatory Biomarkers

**DOI:** 10.3389/fphar.2021.751576

**Published:** 2021-11-12

**Authors:** Acharya Balkrishna, Meenu Tomer, Moumita Manik, Jyotish Srivastava, Rishabh Dev, Swati Haldar, Anurag Varshney

**Affiliations:** ^1^ Drug Discovery and Development Division, Patanjali Research Institute, Governed By Patanjali Research Foundation Trust, Haridwar, India; ^2^ Department of Allied and Applied Sciences, University of Patanjali, Haridwar, India; ^3^ Special Centre for Systems Medicine, Jawaharlal Nehru University, New Delhi, India

**Keywords:** chyawanprash, inflammation, zebrafish, health supplement, medicinal food, nutraceutical

## Abstract

The time-tested Ayurvedic medicinal food, Chyawanprash, has been a part of the Indian diet since ancient times. It is an extremely concentrated mixture of extracts from medicinal herbs and processed minerals, known for its immunity boosting, rejuvenating, and anti-oxidative effects. In this study, we have evaluated the anti-inflammatory potential of Patanjali Special Chyawanprash (PSCP) using the zebrafish model of inflammation. Zebrafish were fed on PSCP-infused pellets at stipulated doses for 13 days before inducing inflammation through lipopolysaccharide (LPS) injection. The test subjects were monitored for inflammatory pathologies like behavioral fever, hyperventilation, skin hemorrhage, locomotory agility, and morphological anomaly. PSCP exerted a strong prophylactic effect on the zebrafish that efficiently protected them from inflammatory manifestations at a human equivalent dose. Expression levels of pro-inflammatory cytokines, like interleukin-6 (IL-6), tumor necrosis factor alpha (TNF-α), and interleukin-1 beta (IL-1β), were also reduced in the LPS-stimulated zebrafish fed on PSCP-infused pellets. Skin hemorrhage, hyperventilation, and loss of caudal fins are characteristics of LPS-induced inflammation in zebrafish. PSCP prophylactically ameliorated skin hemorrhage, restored normal respiration, and prevented loss of caudal fin in inflamed zebrafish. Under *in vitro* conditions, PSCP reduced IL-6 and TNF-α secretion by THP-1 macrophages in a dose-dependent manner by targeting NF-κB signaling, as evident from the secreted embryonic alkaline phosphatase (SEAP) reporter assay. These medicinal benefits of PSCP can be attributed to its constitutional bioactive components. Taken together, these observations provide *in vivo* validation of the anti-inflammatory property and *in vitro* insight into the mode-of-action of Chyawanprash, a traditionally described medicinal food.

## 1 Introduction

Chyawanprash is an amalgamation of two Sanskrit words, “Chyawan” and “Prash”. Chyawan was the name of an ancient Indian sage who, in his quest for enlightenment, underwent strict practices that weakened him leading to rapid aging. In Sanskrit, Chyawan also means “loss” and Prash “foodstuff”. Sage Chyawan was prescribed a health boosting, jam-like tonic to restore his strength, vigor, vitality, and youthfulness, which came to be known as Chyawanprash: the food of sage Chyawan (Sharma et al., 2019). Chyawanprash is indeed a rich health supplement made of several herbs, herbal extracts, and processed minerals, and has been an essential part of the Indian diet for a long time. Health benefits associated with Chyawanprash have been recognized even before the minerals, vitamins, and anti-oxidants became popular as health supplements (Parle and Bansal, 2006).

The ancient claims of Chyawanprash being a medicinal food were proved to be authentic in the light of modern scientific evidence. Several studies have verified that Chyawanprash has several health benefits (Sharma et al., 2019). It is known to improve digestion and metabolism by fortifying liver and kidney functions (Trikam, 1979; Sharma, 2003; Parle and Bansal, 2006). Chyawanprash protects and strengthens the respiratory system. It has been reported to be quite effective as an adjunct to anti-tubercular drugs in not only enhancing the bioavailability of the latter but also in preventing their side effects (Debnath et al., 2012). Chyawanprash is a rich blend of phytocompounds which is believed to be more effective as an anti-oxidant than single phytocompound therapy (Liu, 2004). Besides, Chyawanprash has nootropic potentials (Trikam, 1979; Sharma, 2003; Bansal and Parle, 2011; Parle and Bansal, 2011), can efficiently balance the endocrine system, is a radio- (Jagetia and Baliga, 2004), cyto- (Yadav et al., 2003), and geno-protectant (Uma and Kotasthane, 2014), with anti-mutagenic and anti-carcinogenic potentials (Ghosh et al., 1993; Kohlmeier et al., 1995; Jose et al., 1997). It has been associated with maintenance of favorable lipid profile and glycemic levels (Manjunatha et al., 2001). The herbal ingredients of Chyawanprash have individually been associated with a cardio-protective role (Hashem-Dabaghian et al., 2018). As gathered from the review by Sharma et al. (2019), reports from *in vivo* studies are limited and all these evidences on health benefits of Chyawanprash come mostly from the studies involving the individual herbal component(s) or phytocompound(s) present in it or the observational clinical studies (Sharma et al., 2019). Besides, anti-inflammatory potentials of Chyawanprash have not yet been explored. In the current study, we have used the zebrafish model of inflammation to evaluate the prophylactic anti-inflammatory effect of Patanjali Special Chyawanprash (PSCP). In addition, the *in vivo* observations were recapitulated *in vitro* using human THP-1 macrophages. Furthermore, a modified THP-1 cell line engineered to express the reporter, secreted embryonic alkaline phosphatase (SEAP) under NF-κB stimulation, was used to gain a mechanistic insight into the observed anti-inflammatory effects of PSCP.

Chyawanprash is manufactured by many companies and it has remained a popular health supplement since its entry into the consumer market in 1950 (Sharma et al., 2019). But market trend analysis has shown that consistency and flavor of Chyawanprash not only varies from company to company but also between the different batches from the same company. Chyawanprash is a widely endorsed health supplement (Sharma et al., 2019) with a global market worth ∼7 billion USD (as of April 2020) (Singh, 2020). Therefore, it is essential to maintain high standards in terms of quality of raw materials and finished product of Chyawanprash. So, besides exploring the anti-inflammatory effect, we have conducted a compositional analysis of three independent batches of PSCP. We observed that the variations in the major phytochemical constituents of different batches of PSCP were nominal.

Taken together, our observations revealed that PSCP exhibited an anti-inflammatory property. Protection from inflammation-mediated caudal fin loss in zebrafish subjects fed on PSCP suggested osteoprotective potential. However, further studies are warranted to verify this apparent osteoprotective potential of PSCP. Nevertheless, our findings from this study established the anti-inflammatory attributes of PSCP and identified NF-κB signaling as a molecular mechanism of PSCP.

## 2 Materials and Methods

### 2.1 Source of Test Compound and Reagents

Three independent batches of the test article PSCP [Batch # ADA2000017 (expiry in May 2023), Batch # ADA1900025 (expiry in November 2022), and Batch # ADA2000028 (expiry in September 2023)] were obtained from Patanjali Ayurved Ltd. Haridwar, India. LPS, dexamethasone, and all other chemicals were procured from Sigma Aldrich (St. Louis, MO) unless mentioned otherwise.

### 2.2 Zebrafish Housing, Grouping, Dosing, and Disease Model Establishment

Groups of 24 adult wild type zebrafish were housed under 14 h light and 10 h dark cycle of photoperiod under experimental conditions with housing water temperature maintained at 29°C. The fish were fed with 5 mg per gram body weight of commercial feed as pellets (TetraBit) once per day. All experiments were conducted as per the approved protocol (vide number: 226/Go072020/IAEC) of Institutional Animal Ethics Committee in accordance with the Committee for the Purpose of Control and Supervision of Experiments on Animals (CPCSEA), Government of India. One-year-old zebrafish of either gender weighing 0.5 g, measuring 25–30 mm in length were divided into five groups of 48 each (Table 1). Each group had 24 zebrafish subjects per endpoint. There were two endpoints: one for assessing anti-inflammatory potential of PSCP and the other for survival assay. Oral dosing with positive control, dexamethasone and test article, PSCP were done in the form of infused oral feeds. Stipulated quantities of the compounds were mixed with 4 mg feed extruded into uniform sized pellets. Dexamethasone was administered at the human equivalent dose of 0.08 μg/kg body weight/day (1X-HED-Dexa). PSCP [batch # ADA1900025 (expiry in November 2022)] was administered at the human equivalent dose of 142 μg/kg body weight/day (1X-HED-PSCP) and one-fifth of it at 28 μg/kg body weight/day (0.2X-HED-PSCP). The human equivalent dose of PSCP for zebrafish was determined according to an earlier study by Ducharme et al. (2015), based on its recommended human dose of 10 g/day (Ducharme et al., 2015). Considering the average human body weight to be ∼70 kg, the daily recommended human dose becomes 142 mg/kg. According to Ducharme et al. (2015), the human equivalent dose in zebrafish should be 1,000 times less, which translates into 142 μg/kg (1X-HED-PSCP). Zebrafish maintained on regular feed throughout the experiment constituted the normal control (NC) group. Zebrafish maintained on regular feed but injected with LPS on Day 14 constituted the disease control (DC) group. Zebrafish subjects in 1X-HED-Dexa, 0.2X-HED-PSCP, and 1X-HED-PSCP groups received respective treatments until Day 13 and were injected with LPS on Day 14. In order to induce inflammation, 3.33 μg/μl of LPS was injected using a Hamilton syringe between the lateral line and the anal pore. Control groups were injected with 1% saline. Zebrafish were anesthetized by sequentially transferring them to 17°C and 12°C water. Screening was done 24 h after induction of inflammation.

**TABLE 1:** Group, diet, dosage, and inflammation status details.

Name of the group	Group size^f^	Diet details	Dosage (µg/kg BW^i^/day)^j^	LPS induced inflammation
NC^a^	48	Regular pellet	na	✗
DC^b^	48		na	✓
1X-HED^c^-Dexa^d^	48	Dexamethasone^g^ infused pellet	0.08	✓
0.2X-HED-PSCP^e^	48	PSCP^h^ infused pellet	28	✓
1X-HED-PSCP	48		142	✓

a Normal Control.

b Disease Control.

c Human Equivalent Dose.

d Dexamethasone.

e Patanjali Special Chyawanprash.

f Each group had 24 subjects/endpoint. There were two endpoints.

g,h Human Equivalent Dose of Dexamethasone and PSCP, are 6 mg/day^g^ and 10 g/day^h^, respectively.

i Body Weight.

j Zebrafish weighing 0.5 g were included in the study.

### 2.3 Endpoint Measurements for Screening

#### 2.3.1 Behavioral Fever

Zebrafish being an ectotherm exhibits behavior fever as an adaptive immunity. To study the behavior fever, an experimental glass tank was set up interconnected chambers at three different temperatures (23°C, 29°C, and 37°C). The temperature gradient in the tank was maintained by continuous heating and cooling from flanking chambers at 18 and 40°C at its anterior and posterior ends, respectively. The zebrafish subjects from the respective study groups were introduced individually into the chamber at 29°C with sufficient time to choose the temperature that supports the body temperature. The time spent by the fish in the temperature gradient chamber was noted for consecutive 3 min with a stopwatch, and observed findings were represented as the heat map.

#### 2.3.2 Behavioral Phenotype

The fish were introduced from the housing tank to the study tank and were allowed to acclimatize for 3 min. The swim velocity (mm/s), path profile, and the number of bends the fish took over an observation period of 3 min were recorded using a DSLR camera (D3100 Nikon Corporation, Tokyo, Japan). The results were analyzed using Mtrack2 Version: v1.52n plugin of Image J software (Meijering et al., 2012). In order to eliminate the circadian-induced bias, the assay was carried out during the light cycle (6 am–5 pm).

#### 2.3.3 Heart Rate Measurement

Fish were introduced into the cognitive tank and were allowed to acclimatize until the erratic movement ceased. Subsequently, the operculum movement was recorded using a DSLR camera for a duration of 3 min. Based on the value obtained, the number of heartbeats per minute was derived from the operculum movement. Four operculum movement is considered equal to one heartbeat.

#### 2.3.4 Phenotype Imaging

The zebrafish subjects in different study groups were observed for skin hemorrhages. Microscopic images of the hemorrhagic spots were captured using stereomicroscope at 10X magnification using CZM4 stereomicroscope (Labomed, Los Angeles, CA). Hemorrhagic spots and area of tissue degeneration were quantified using ImageJ software (National Institute of Health, MA).

#### 2.3.5 Semi-Quantitative Estimation of Expression of Inflammatory Markers

QIAamp RNA Blood Mini Kit (Qiagen, Hilden, Germany) was used to extract total RNA from peripheral blood. After DNase I (New England Biolabs, Ipswich, MA) treatment, cDNA was synthesized, according to manufacturer’s protocol, from 1 µg of total RNA using oligo (dT) primer and SuperScript II reverse transcriptase (Invitrogen, Carlsbad, CA). Thereafter, semi-quantitative PCR was carried out using REDTaq® ReadyMix™ PCR Reaction Mix (Sigma Aldrich, St. Louis, MO) following the manufacturer’s protocols. There were 30 cycles of PCR amplifications carried out in Applied Biosystems GeneAmp PCR System 9,700 (Foster City, CA). The following primer sequences were used: C-reactive protein (CRP) (Forward 5′-AAA​ACC​TGC​TGA​ATC​GGA​CT-3’; T_m_: 62.6°C and reverse 5′- ACT​TCG​GTA​GCC​GCT​AAT​GT -3’; T_m_: 62.5°C), interleukin-6 (IL-6) (Forward 5′-AGC​GTC​TTC​ACC​GAA​GTC​TG-3’; T_m_: 64.6°C and reverse 5′- GGT​TGG​TTG​GAG​GAT​CCA​GG -3’; T_m_: 67.8°C), interleukin-1beta (IL-1β) (Forward 5′- TTC​CCC​AAG​TGC​TGC​TTA​TT -3’; T_m_: 63.4°C and reverse 5′- AAG​TTA​AAA​CCG​CTG​TGG​TCA-3’; T_m_: 63.3°C) and tumor necrosis factor (TNF-α) (Forward 5′- ACC​AGG​CCT​TTT​CTT​CAG​GT -3’; T_m_: 63.8°C and reverse 5′- GCA​TGG​CTC​ATA​AGC​ACT​TGT​T-3’; T_m_: 65.1°C). GAPDH (Forward 5′- GTG​TAG​GCG​TGG​ACT​GTG​GT -3’; T_m_: 65.1°C and reverse 5′- TGG​GAG​TCA​ACC​AGG​ACA​AAT​A-3’; T_m_: 65.3°C) was included as internal reference. The amplicons were resolved using agarose gel electrophoresis, and fluorescence was quantified by ImageJ software from National Institute of Health (MA) (Schneider et al., 2012).

#### 2.3.6 Kaplan-Meier Survival Curve

Mortality was counted daily to estimate the survival rate over therapeutic intervention among the zebrafish subjects in different study groups.

#### 2.3.7 Evaluation of Cytotoxicity, Effect on *in vitro* Cytokine Response and NF-κB Signaling

Cytosafety and anti-inflammatory effect of PSCP was evaluated in THP-1 cells (RRID: CVCL_0006), procured from ATCC recognized cell repository at National Centre for Cell Sciences (NCCS), Pune, India, and cultured in RPMI-1640 media (cat #31800, Gibco, Amarillo, TX), supplemented with 10% heat-inactivated fetal bovine serum (cat # RM9955, Himedia, Mumbai, India) in the presence of penicillin–streptomycin (100 U/ml) (cat #P4333, Sigma, St. Louis, MO). These assays were conducted as described earlier (Balkrishna et al., 2020). THP-1 cells, seeded at a density of 5 × 10^4^ cells/well, were differentiated into macrophages with 20 ng/ml phorbol 12-myristate 13-acetate (PMA) (cat #K63916, Alfa Aesar, Haverhill, MA) for 16 h followed by a rest period of 6 days. Cells were pre-treated with different concentrations (3, 10, 30, 100, 300, and 1,000 μg/ml) of PSCP for 24 h before inducing inflammation with LPS (500 ng/ml). LPS treated cells were co-treated with above mentioned concentrations of PSCP for the entire duration of 6 h of inflammation induction. Thereafter, levels of secreted interleukin-6 (IL-6) (cat #555220) and tumor necrosis factor alpha (TNF-α) (cat #555212) were measured in the cell supernatants using ELISA kits from BD Biosciences (Franklin Lakes, NJ) following the manufacturer’s protocol. Viability of the same cells was determined through fluorescence at 560 nm (excitation) and 590 nm (emission) from Alamar blue (15 μg/ml) (cat #TC235-1G, Himedia, Mumbai, India) staining. The effect of PSCP on NF-κB signaling was evaluated using THP1-Blue™ NF-κB (cat # thp-nfkb), SEAP reporter cells from InivoGen (San Diego, CA). These cells were grown in RPMI containing HEPES (25 mM, MB016, Himedia, Mumbai, India), penicillin–streptomycin (100 U/ml), 100 μg/ml Normocin (cat # ant-nr, InvivoGen), and 10% heat inactivated FBS. For SEAP reporter assay, 1 × 10^5^ THP1-Blue™ NF-κB cells/well were seeded in 96-well plate and treated with different concentrations of PSCP (3, 10, 30, 100, 300, and 100 μg/ml) for 24 h before inducing NF-κB transcriptional activity with 100 ng/ml of LPS for another 6 h. The cells were co-treated with corresponding concentrations of PSCP during LPS induction. SEAP levels in the supernatant were measured using QUANTI-Blue™ solution (InvivoGen) as per manufacturer’s protocol. All cells were maintained in a humidified incubator at 37°C with 5% CO_2_.

#### 2.3.8 Data Analysis

All graphs were plotted using GraphPad Prism 8.0 software (La Jolla, CA) and data displayed as mean ± SEM. Statistical significances of the observations were analyzed at 95% confidence level using built-in options of the GraphPad Prism software. The survival was analyzed through a Kaplan-Meier plot.

### 2.4 Compositional Analysis

#### 2.4.1 Standard and Sample Preparation

There was 100 μg/ml stock solutions of all marker compounds prepared from which required working concentrations were diluted for further use. There was 500 mg of PSCP from each batch dispersed in 10 ml with methanol through sonication and centrifuged at 10,000 rpm for 5 min for a clear supernatant which was filtered through 0.45 µm nylon filter before using for further analysis.

#### 2.4.2 HPTLC Fingerprinting

Camag HPTLC system equipped with an automatic TLC sampler (ATS4), TLC scanner 4, and integrated software Win-CATS was used for analysis HPTLC fingerprinting. HPTLC was performed on a precoated silica gel 60F_254_ plate. TLC plate development was carried out in a camag twin trough chamber, which was presaturated for 15 min with mobile phases, toluene: ethyl acetate: formic acid (9: 10: 1 v/v/v) for Gallic acid, Piperine, Cinnamic acid, and Eugenol; ethyl acetate: formic acid: acetic acid: water (10: 1: 1: 2.3 v/v/v) for Glycyrrhizin; and ethyl acetate: toluene: formic acid: methanol (9: 9: 2:0.6 v/v/v/v) for Ellagic acid. TLC plates were dried using an air dryer and scanned for quantitative analysis at 254 nm for Glycyrrhizin, 280 nm for Gallic acid, Cinnamic acid, Eugenol, and Ellagic acid, and 340 nm for Piperine.

#### 2.4.3 HPLC Analysis

The quantification of marker compounds was performed by HPLC (Prominence-i LC-2030c 3D Plus, Shimadzu, Japan). Separation was achieved using a Shodex C18-4E (5 μm, 4.6 × 250 mm) column subjected to binary gradient elution. The elution was carried out at a flow rate of 1.0 ml/min using mobile phase A (0.1% orthophosphoric acid in water) and mobile phase B (0.1% orthophosphoric acid in acetonitrile: water:88:12). pH of both the mobile phases was adjusted to 2.5 with diethylamine. Column temperature was maintained at 35.0°C during the analysis. There was 10 µl of test solution injected and chromatogram was recorded at 278 nm (for Gallic acid, Corilagin, Chebulagic acid, Cinnamic acid, Eugenol, and Piperine) and at 250 nm (for Glycyrrhizin and Ellagic acid).

## 3 Results

### 3.1 Study Design, Prophylactic Inflammatory Model Set Up and Survival Assay

**FIGURE 1:**
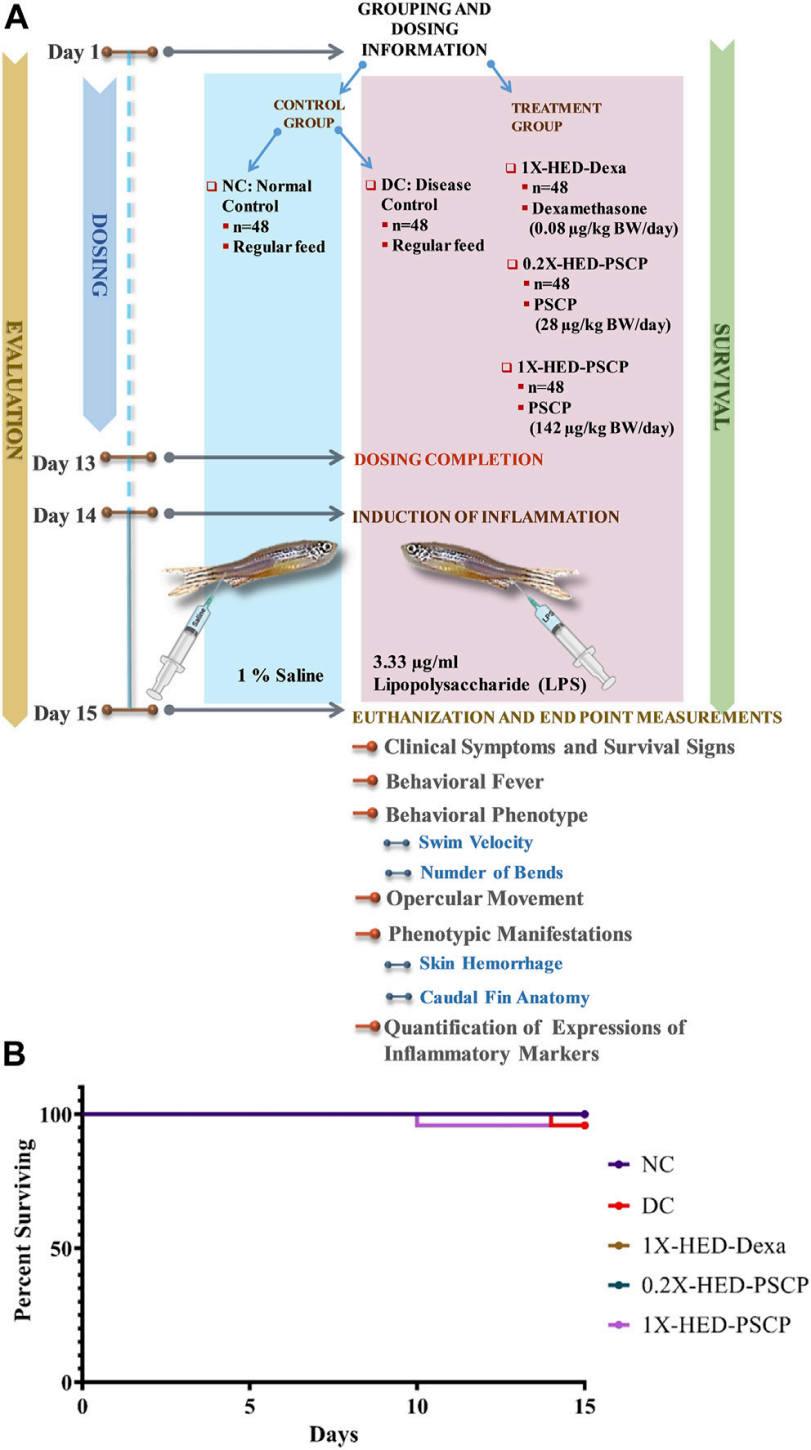
Study plan and effect of PSCP on zebrafish survival. **(A)** Schematic illustrates the entire study plan. PSCP as an anti-inflammatory agent was evaluated. Its effects on behavioral, anatomical, and physiological manifestations were carried out in parallel with assessing its consequences on the survival of the zebrafish. The schematic details the timeline of the experiment, groups, and administered doses. The study was carried out for 15 days, starting with grouping and dosing on Day 1. Five groups, namely, normal control (NC), disease control (DC) under control category, and 1X-HED-Dexa, 0.2X-HED-PSCP, and 1X-HED-PSCP under treatment category were formed. Each group consisted of 48 fish; 24 of which were dedicated for evaluating behavioral, anatomical, and physiological manifestations of PSCP feeding, whereas the remaining 24 fish in each group were followed for survival analysis. Zebrafish in the NC and DC groups were fed regular pellets, those in the 1X-HED-Dexa group received dexamethasone enriched pellets at 0.08 μg/kg body weight/day, which is the human equivalent dose (HED) of dexamethasone for zebrafish. Zebrafish in groups 0.2X-HED-PSCP and 1X-HED-PSCP were fed with pellets enriched with PSCP at 28 and 142 μg/kg body weight/day, respectively, which was one-fifth of the human equivalent dose, that is, 0.2X-HED and the human equivalent dose, that is, 1X-HED. Treatments were administered for 13 days followed by induction of inflammation through lipopolysaccharide (LPS) injection on Day 14. Zebrafish of all except the NC group were injected with LPS. The endpoint parameters, namely, behavioral fever, swim velocity, number of bends taken by each fish during swimming, opercular movement, anatomical effects, like skin hemorrhage and morphological anomaly of caudal fins and immunological responses such as alterations in expressions of inflammatory markers, such as interleukin-6 (IL-6), C-reactive protein (CRP), tumor necrosis factor alpha (TNF-α), and interleukin 1-beta (IL-1β), were measured on Day 15. **(B)** Survival of the zebrafish included in the study is represented as a Kaplan-Meier curve.

Chyawanprash is a traditionally recognized bioactive medicinal food. Therefore, this study aims at evaluating its prophylactic effects as a moderator of inflammatory reactions. Therefore, experimental design involved pre-treatment of zebrafish with PSCP followed by induction of inflammation with lipopolysaccharide (LPS) (Figure 1A). The zebrafish were divided into five groups, namely, NC or normal control, DC or disease control, 1X-HED-Dexa receiving human equivalent dose (HED) of dexamethasone (taken as positive control), 0.2X-HED-PSCP fed with 0.2 times human equivalent dose of PSCP and 1X-HED-PSCP that received its human equivalent dose. The zebrafish in NC and DC groups were fed on regular pellets whereas the feeds for the other three groups were enriched with the respective compounds in the designated doses. The details of diet and dosage and inflammation statuses of zebrafish in different groups are summarized in Table 1. The fish received the designated feeds daily until Day 13. On Day 14, inflammation was induced with LPS in all fish, except those belonging to the NC group. On the following day, the fish were euthanized and end point parameters for inflammation in zebrafish were evaluated. These included behavioral fever, physical activeness (in the form of swim velocity and the number of bends taken by the fish during swimming), hyperventilation (measured as the rate of opercular movements), anatomical changes such as skin hemorrhage and morphological alterations, like structural anomaly of caudal fin. In parallel to this screening study, a survival assay was also conducted until Day 15 and observation represented as Kaplan-Meier (K-M) plot (Figure 1B). From the K-M plot, 100% survival is evident among the fish of the NC group. The survival dropped to 95.8% in the DC group, but the observation was not statistically significant when compared to the NC group. Dexamethasone enriched diet did not affect the survival of the zebrafish. The statistically insignificant mortality observed in the 1X-HED-PSCP treated group is similar to that seen for the DC group and, therefore, cannot be correlated to the treatment. Besides, both the PSCP treated groups showing the same survival percentages supports our previous argument. Thus, the decided doses were safe for our study model.

### 3.2 Pre-Treatment with Patanjali Special Chyawanprash Prevented Behavioral Fever Symptoms in Zebrafish

**FIGURE 2:**
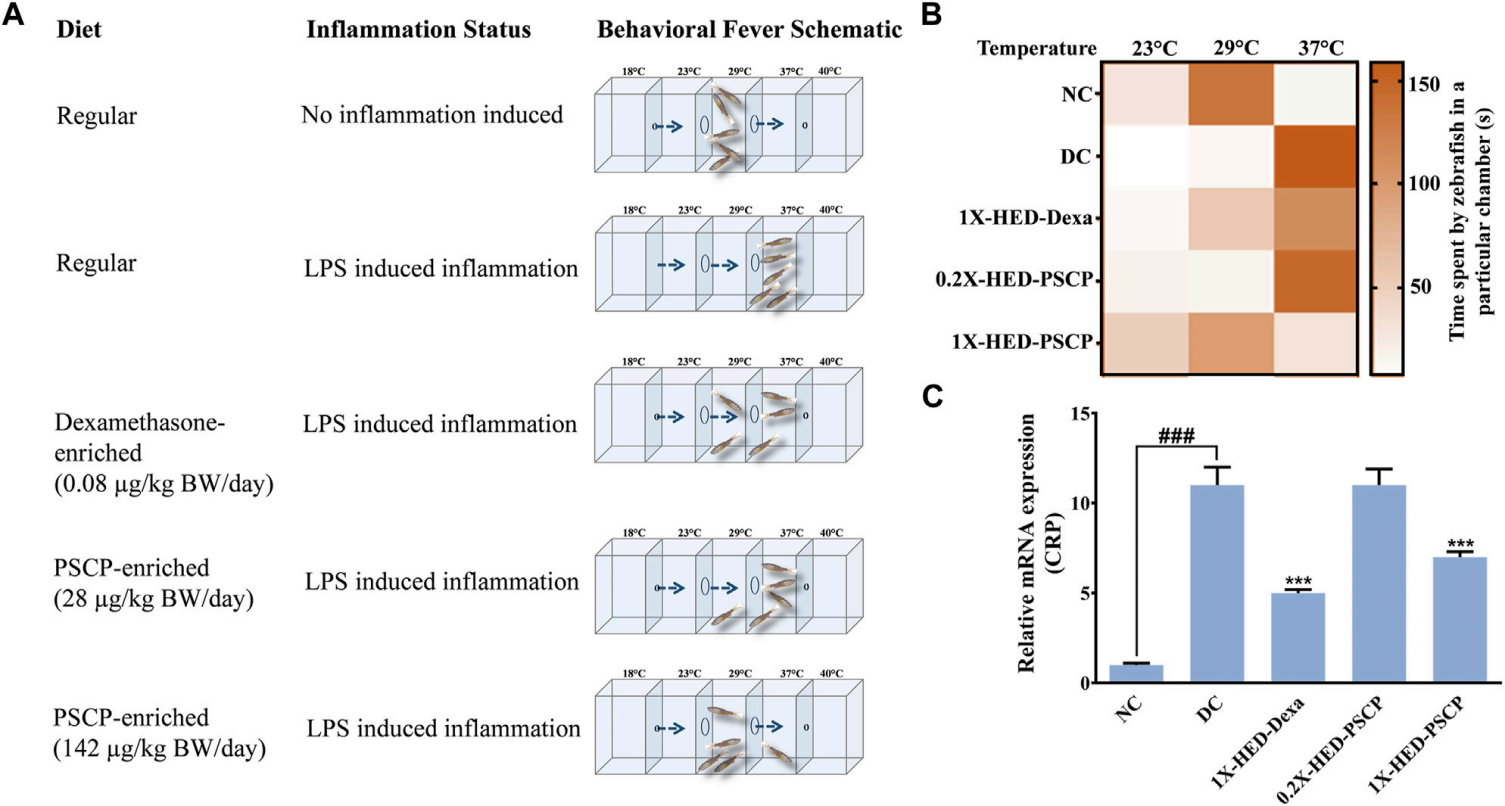
Pre-treatment with PSCP prevents LPS induced behavioral fever in zebrafish. **(A)** Schematic summarizes the diet provided to the zebrafish in each group, the status of inflammation in these fish and the expected outcome in terms of behavioral fever for each combination of diet and status of inflammation. The water tanks have three chambers at 23, 29, and 37°C in the middle with flanking chambers at 18 and 40°C at the anterior and distal ends of the tank, respectively. LPS induced inflammation causes the internal body temperature of zebrafish to rise, prompting them to migrate toward a higher temperature (which otherwise confines to water at 29°C) thereby depicting behavioral fever. A prophylactic effect of PSCP is captured through cartoons illustrating more time spent by fish in a chamber with water at 29°C. Dexamethasone treated zebrafish also spent more time in a chamber with water at 29°C. **(B)** Time spent in different temperature zones by zebrafish in each group was recorded and represented as a heat map. The darker shade of the orange hue depicts the longer time duration spent by a fish in a given temperature zone as conveyed through the palate bar on the side. **(C)** Rescuing from inflammation induced behavioral fever is biochemically confirmed through the corresponding changes in the mRNA levels of C-reactive protein (CRP). Data represented is mean ± SEM. Statistical significance, evaluated through one-way ANOVA followed by Tukey’s post-hoc test is indicated as either ### or *** for *p* < 0.001 depending on whether compared to the NC or the DC group.

Zebrafish being ectothermic exhibits behavioral fever as a symptom of adaptive immune reactions to inflammation. Fish suffering from inflammation have raised body temperatures which prompt them to migrate toward warmer water. The ambient water temperature for regular zebrafish is 29°C. As depicted in Figure 2A, the specialized tanks for assessing behavioral fever in zebrafish consist of three interconnected chambers in which the water is maintained at 23, 29, and 37°C. The flanking chambers have cooler (18°C) and warmer (40°C) water and are connected to cooling and heating facilities, respectively. The water is allowed to flow freely through these chambers to ensure the maintenance of this multi-temperature arrangement throughout the experiment. However, the flanking chambers are non-accessible to the fish. After a fish is introduced into the assessment tank, it is allowed to equilibrate with its surroundings before recording the duration of time it spends in each temperature zone. Each fish was monitored for 3 min. The recorded time durations are represented as a heat map with a gradient of orange palette used to indicate the stay times. The darker the shade of orange, the longer the time spent, as shown in the index bar with the time marked in minutes (Figure 2B). The zebrafish in the NC group that were fed with regular pellets and did not have any inflammation spent the entire duration of 3 min in the chamber with water at 29°C (Figures 2A,B, first rows). Without any prophylactic protection, the members of the DC group upon developing inflammation, exhibited behavioral fever as evident from their flocking in the chamber of the water tank maintained at 37°C (Figures 2A,B, second rows). Fish in the 1X-HED-Dexa group that received the human equivalent dose of dexamethasone daily for 13 days, were observed to spend a part of the 3 min duration of monitoring at 29°C, although 37°C was apparently preferred more (Figures 2A,B, third rows). When the zebrafish from the 0.2X-HED-PSCP group, pre-treated with PSCP at a dose 5 times lesser than the human equivalent one, were exposed to inflammation, they spent most of the time at 37°C (Figures 2A,B, fourth rows). This suggested that the low dose of PSCP did not confer any prophylactic effect against inflammation in these fish. However, at human equivalent dose, PSCP exhibited significant prevention of behavioral fever among the members of the 1X-HED-PSCP group (Figures 2A,B, fifth rows). Fever is a symptomatic manifestation of infection, in this case reproduced in zebrafish by using LPS. Increased serum level of C-reactive protein (CRP) is the biochemical indicator of such infection associated inflammation (Sproston and Ashworth, 2018). Therefore, CRP levels in the zebrafish subjects from different groups were assessed through semi-quantitative RT-PCR. It was observed that the average relative CRP mRNA level was significantly increased (11-fold) after LPS injection in the DC group. After dexamethasone treatment at a human equivalent dose, the relative CRP mRNA level was reduced by 2.2-fold in the 1X-HED-Dexa group compared to the DC group. PSCP at a low dose (one-fifth of the human equivalent dose) did not affect the CRP mRNA level in the 0.2X-HED-PSCP group compared to the DC group (both showing an 11-fold increase relative to the NC group). However, at a human equivalent dose, PSCP in 1X-HED-PSCP group reduced CRP mRNA level by 1.6-fold compared to the DC group (Figure 2C). Taken together, these observations conclusively prove that PSCP, at a human equivalent dose, when fed for a period of 13 days, can prevent fever induced by inflammation in zebrafish.

### 3.3 Patanjali Special Chyawanprash Efficiently Staves Off Inflammation Induced Locomotory Distress in Zebrafish

**FIGURE 3:**
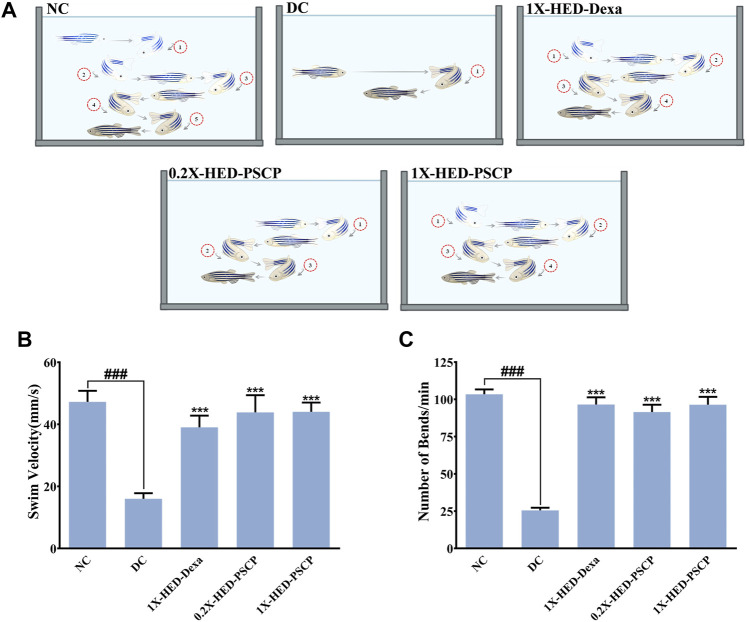
PSCP enriched diet prevents loss of physical activeness in zebrafish due to inflammation. **(A)** Pictorial depiction of the effect of different pre-treatments and subsequent induction of inflammation in zebrafish on their swim velocity and locomotory agility discerned as taken by a fish during swimming for a given duration (in this case, 1 min). Swim trajectories are shown with a combination of different grades of fading zebrafish pictures and arrows: lighter the fish meaning farther in the past that part of the trajectory has been traversed by the fish. Each bend has been numbered to convey the lost swim agility due to inflammation as a smaller number of observed bends taken by an inflamed fish during swimming and prevention of the same due to pre-treatments with PSCP and positive control dexamethasone. **(B,C)** Swim velocity **(B)** and number of bends **(C)** taken by each zebrafish while swimming for a given duration were determined and represented graphically. Data shown is mean ± SEM. Statistical significance of the observed difference among the means of difference groups was evaluated through one-way ANOVA followed by Tukey’s post-hoc test and indicated as ### or *** for *p* < 0.001 depending on whether compared with the mean of NC or DC.

Adult zebrafish exhibit reduced locomotion as a response to inflammatory challenge. This behavior of zebrafish was used to assess the prophylactic anti-inflammatory effect of PSCP (Campos-Sánchez and Esteban, 2021). This is also an indication of the physical activeness that gets affected upon inflammation. Locomotory agility of the zebrafish in different groups was assessed from swim velocity and the number of bends the fish took while swimming for a certain period. The observed outcomes of this assessment are represented pictorially in Figure 3A. Reduction in swim velocity and agility are depicted as shorter and less labyrinthine trajectories. The healthy zebrafish on a normal diet without any inflammation were noted to be fast swimmers, moving at an average velocity of 47.26 ± 16.94 mm/s (Figure 3B). These fish were quite agile too as evident from an average of 104 ± 15.32 bends they took in a minute while swimming (Figure 3C). The members of the DC group who did not receive any pre-treatment but were exposed to inflammatory challenge in the form of LPS injection responded with significantly reduced swim velocity (16.04 ± 9 mm/s; *p* < 0.001) as compared to the NC group. These DC group zebrafish also took a lesser amount of bends per minute while swimming (26 ± 8.21 bends/min vs 104 ± 15.32 bends/min in the NC group, *p* < 0.001) (Figures 3B,C). As expected, pre-treatment with the positive control, dexamethasone, moderated locomotory distress efficiently. Zebrafish in the 1X-HED-Dexa group exhibited an average swim velocity of 39 ± 18.07 mm/s which was significantly high when compared to the DC group (*p* < 0.001). They also took almost as many bends as the fish in the NC group in a minute (97 ± 23.14 bends/min), which was 3.7 times more relative to the DC group, offering a statistically significant improvement in the swim agility (Figures 3B,C). Pre-treatment with PSCP at low and high doses was equally effective in preventing locomotory distress in zebrafish due to inflammation. The average swim velocities in the 0.2X-HED-PSCP and 1X-HED-PSCP groups were 44 ± 26.41 and 44 ± 14.3 mm/s, which were significantly higher than that of the DC group (*p* < 0.001) and were comparable to the NC group. These fish also exhibited locomotory prowess equivalent to the healthy subjects of the NC group (91 ± 23.7 and 96 ± 24.9 bends/min for the 0.2X-HED-PSCP and 1X-HED-PSCP groups, respectively) (Figures 3B,C).

### 3.4 Patanjali Special Chyawanprash Prevented Inflammation-Mediated Increase in Heartbeat of Zebrafish

**FIGURE 4:**
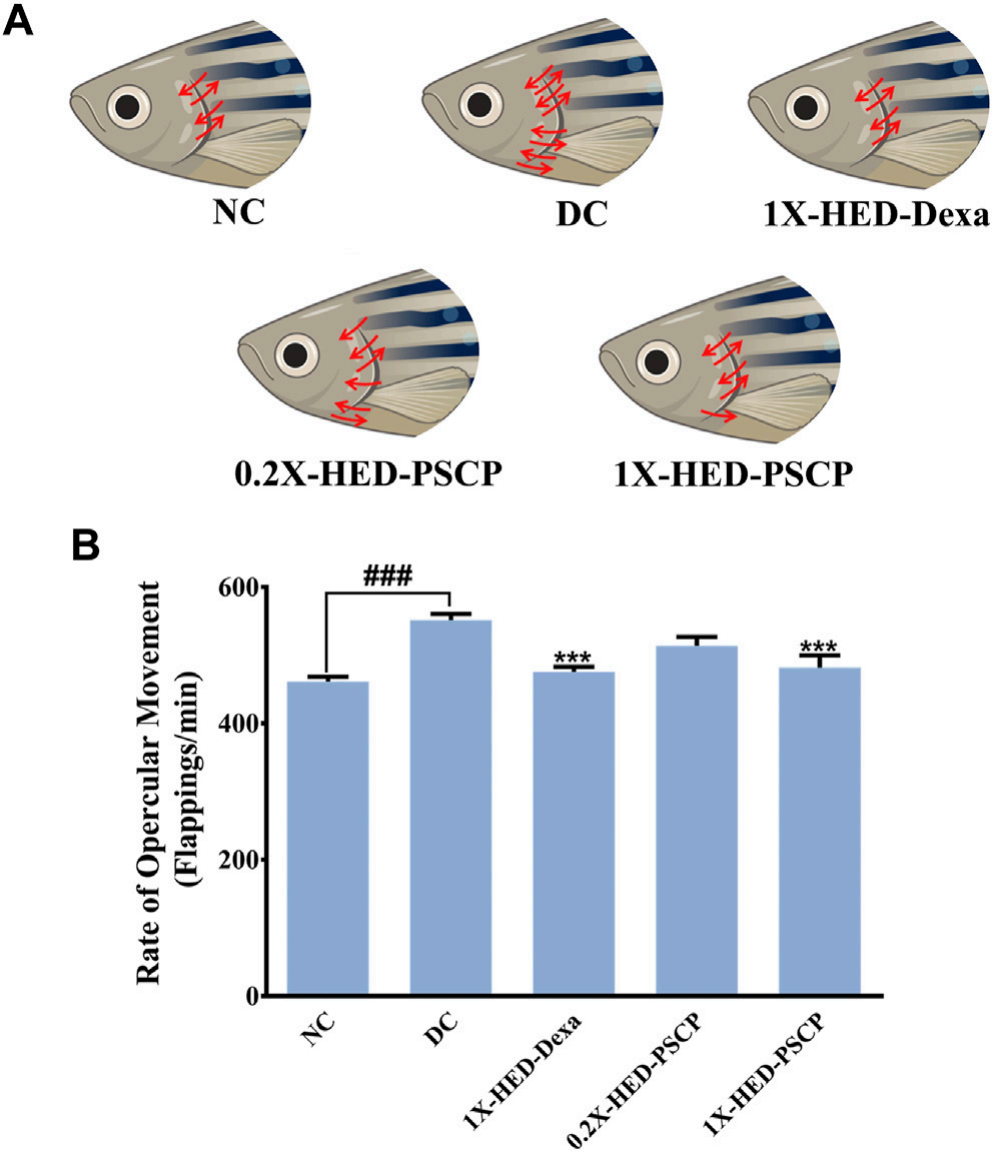
PSCP prevents inflammation induced hyperventilation in zebrafish. **(A)** Hyperventilation in zebrafish is depicted pictorially through increased opercular movements, indicated with red arrows in either direction over the fish operculum. Number of arrows is proportional to the degree of hyperventilation observed in the zebrafish of that particular group. **(B)** Opercular movements (flappings/min) were noted and represented graphically as mean ± SEM. One-way ANOVA with Tukey’s post-hoc test was used to determine the statistical significance of the observation and indicated with either ### or *** for *p* < 0.001 depending on whether the comparison is with mean of NC or DC.

Exposure to LPS induces hypoxia in zebrafish (Philip et al., 2017) and concomitant hyperventilation (Vulesevic et al., 2006), evident as increased breathing measurable through the rate of opercular flappings. Heartbeats of zebrafish are indirectly measured as the rate of opercular movements. Four opercular flappings are taken as one heartbeat (Weintraub and Mackay, 1975; Mendez-Sanchez and Burggren, 2017). Status of opercular movements in different groups are represented pictorially in Figure 4A. The arrows over the fish operculum depict movement and their numbers are representative of the rate of opercular movement observed in the particular group. As contemplated, the zebrafish in the DC group when injected with LPS, exhibited an increase in the average rate of opercular movement (551.8 ± 8.8 flappings/min) as compared to an average of 461.3 ± 7.1 flappings/min in the NC group. Pre-treatment with dexamethasone reduced this rate to 475.8 ± 7.2 flappings/min in the 1X-HED-Dexa group. Zebrafish in the 0.2X-HED-PSCP and 1X-HED-PSCP groups, respectively, receiving one-fifth of the human equivalent dose and the human equivalent dose of PSCP, recorded reduced rates of opercular movements. The average rate of opercular movement in the 0.2X-HED-PSCP group was 513.8 ± 13.1 flappings/min. The average rate of opercular movement was further reduced in the 1X-HED-PSCP group to 481.8 ± 17.9 flappings/min, which was comparable to that found in the NC group (Figure 4B).

### 3.5 Lipopolysaccharide Induced Pro-Inflammatory Cytokine Response Was Moderated by Patanjali Special Chyawanprash

**FIGURE 5:**
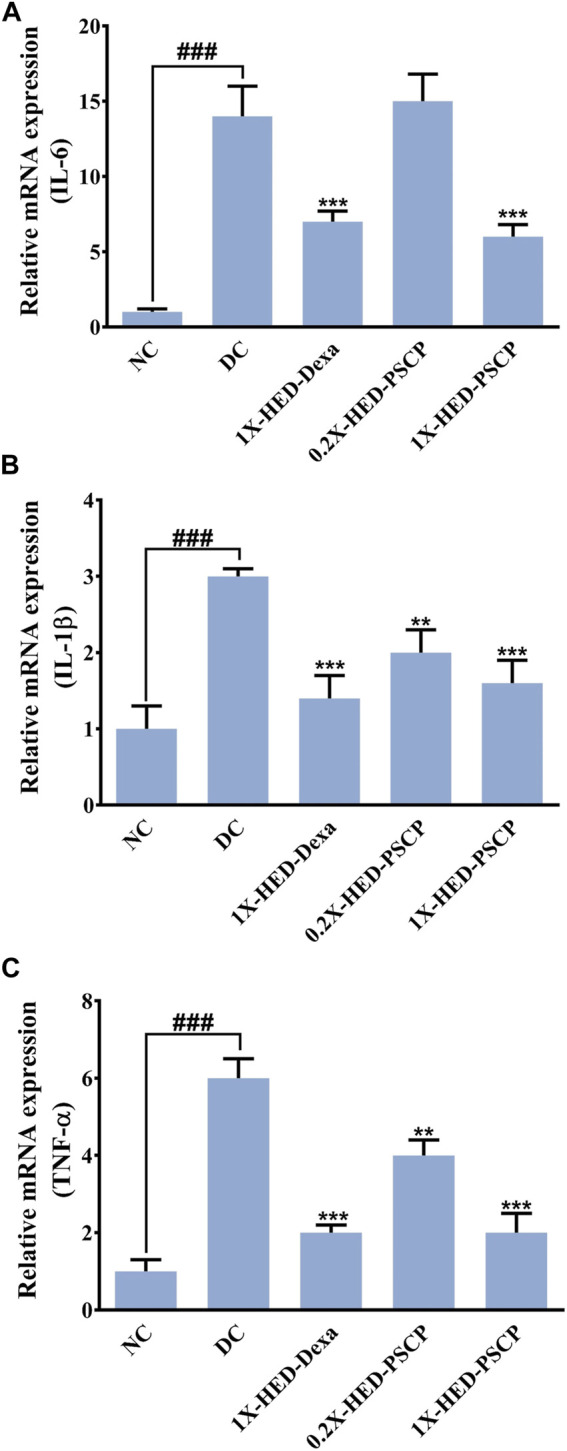
PSCP moderates expressions of pro-inflammatory cytokines in zebrafish. **(A-D)** Alterations in expressions of inflammatory markers, namely, IL-6 **(A)**, IL-1β **(B)**, and TNF-α **(C)** and in zebrafish in response to LPS induced inflammation were evaluated through semi-quantitative RT-PCR and represented as relative mRNA expressions. Data represented is mean ± SEM. Statistical significance was evaluated through one-way ANOVA followed by Tukey’s post-hoc test and indicated as ### for *p* < 0.001 when compared to NC group. Statistical significances were shown as ** and *** for *p* < 0.0.01 and 0.001, respectively, when the comparison was done with mean of the DC group.

LPS induces cytokine response which was used as a parameter to evaluate the anti-inflammatory effect of PSCP. mRNA levels of pro-inflammatory cytokines, interleukin-6 (IL-6), interleukin-1β (IL-1β), and tumor necrosis factor-α (TNF-α) were measured through semi-quantitative RT-PCR in different groups. In comparison to the NC group, mRNA levels of IL-6, IL-1β, and TNF-α were increased by 14-, 3-, and 6-fold, respectively, in the DC group in which the zebrafish subjects were injected with LPS but were not given any remedial treatment. In the positive control 1X-HED-Dexa group, the mRNA levels of IL-6, IL-1β, and TNF-α were reduced by ∼ twofold. A lower dose of PSCP did not reduce the levels of pro-inflammatory cytokines effectively. However, reduction similar to the positive control (2.3- and 1.9-folds for IL-6 and IL-1β, respectively) was observed in the 1X-HED-PSCP group where the zebrafish were treated with the human equivalent dose of PSCP. This reduction was found to be threefold in the case of TNF-α (Figures 5A–C). Thus, feeding PSCP helped in reducing the LPS induced inflammatory response in zebrafish.

### 3.6 Patanjali Special Chyawanprash Effectively Intercepts Lipopolysaccharide Mediated Physiological Anomaly and Morphological Deformity

**FIGURE 6:**
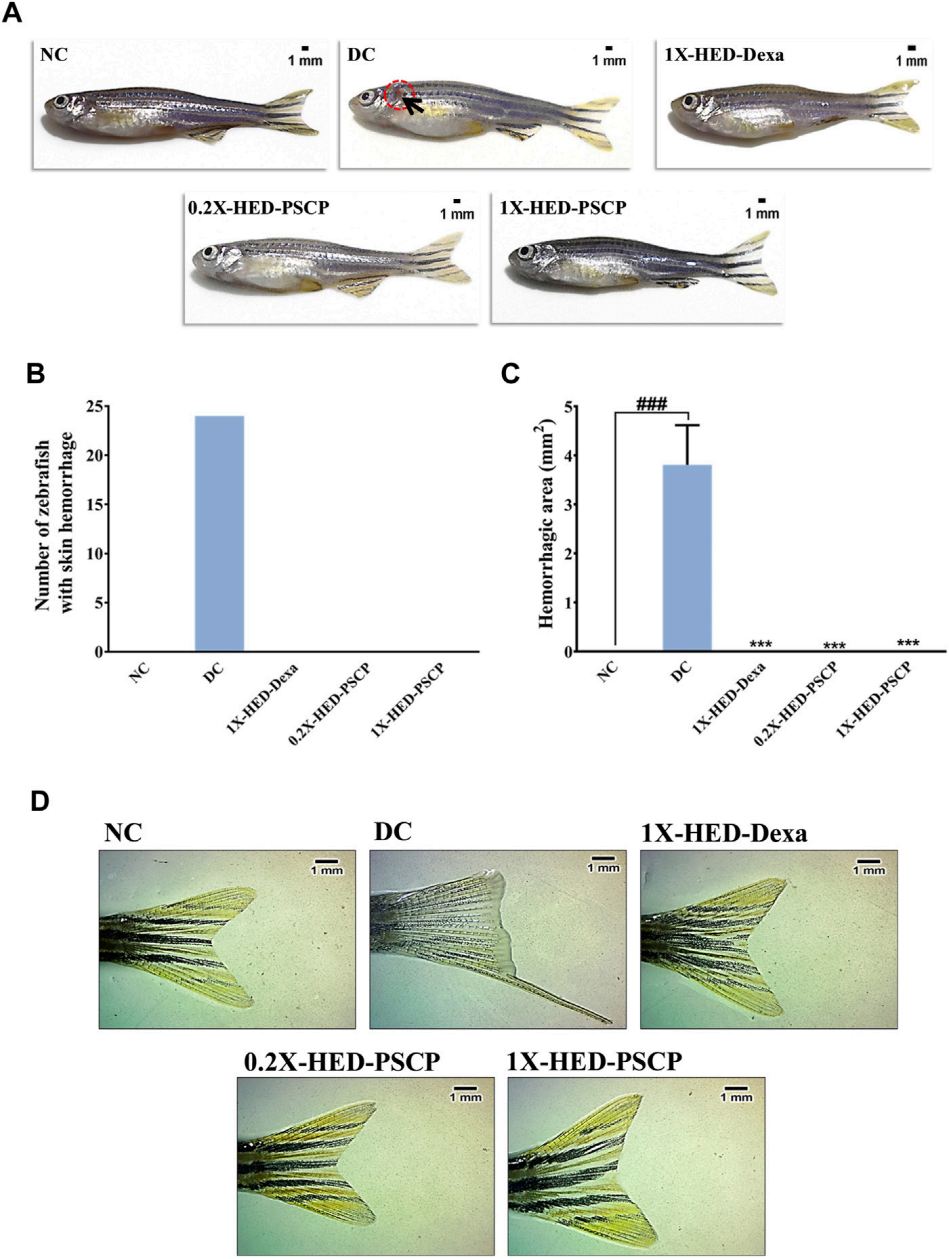
Inflammation induced anatomical anomalies in zebrafish are averted by PSCP. **(A)** Representative digital images of zebrafish from each group to show the presence of skin hemorrhage (encircled in open red circle and indicated with black arrow) or its lack thereof. **(B)** The occurrences of skin hemorrhages were counted for each group and represented graphically. **(C)** The areas of damaged tissue owing to skin hemorrhage were also determined and shown graphically as mean ± SEM. Statistical significance of the observation was confirmed through one-way ANOVA and Tukey’s post-hoc test and indicated as ### when compared to the NC group or as *** in comparison to the DC group for *p* < 0.001. **(D)** Effect of inflammation on caudal fin morphology and prevention of such effect by treatments are shown as representative digital images of caudal fins of a fish from each group.

Inflammation dysregulates blood coagulation (Esmon, 2005). LPS induced activation of blood coagulation cascade is reported in humans (Pernerstorfer et al., 1999; Koch et al., 2009). Zebrafish has human orthologs of the factors involved in blood coagulation and, therefore, disorders of blood coagulation have been successfully modelled on zebrafish (Kretz et al., 2015; Jagadeeswaran et al., 2016). Thus, we used skin hemorrhage as an outcome to evaluate the anti-inflammatory efficacy of PSCP. The subjects of the NC group, which received a regular diet and were not injected with LPS, did not develop skin hemorrhage (Figures 6A–C). Induction of inflammation through the endotoxin LPS resulted in visible signs of skin hemorrhage near the gill in the zebrafish subjects of the DC group, indicating inflammation (Figure 6A). In fact, clinical signs of skin hemorrhage were observed in all the subjects of the DC group (Figures 6B,C). Zebrafish subjects of the 1X-HED-Dexa group receiving dexamethasone at a human equivalent dose of 0.08 μg/kg body weight/day exhibited no clinical signs of skin hemorrhage (Figures 6A–C). Likewise, skin hemorrhage was not visible in the zebrafish subjects of 0.2X-HED-PSCP and 1X-HED-PSCP, fed on 28 and 142 μg/kg body weight/day of PSCP, respectively (Figures 6A–C). From these observations, we concluded that PSCP was effective in rescuing the zebrafish from inflammation driven skin hemorrhage.

Caudal fin loss is one of the pathological features of LPS induced inflammation in zebrafish (Philip et al., 2017). We observed that zebrafish subjects in the DC group, which were injected with LPS (recapitulating bacterial infection), exhibited partial caudal fin loss (Figure 6D, panel DC). Such morphological deformity was clearly absent in the subjects of the NC group, without infection (Figure 6D, panel NC). Dexamethasone treatment prevented caudal fin deformity (Figure 6D, panel 1X-HED-Dexa). We observed normal caudal fin morphology with sharp edges and well-defined fin architecture in the zebrafish subjects of 0.2X-HED-PSCP and 1X-HED-PSCP groups, which were fed with different concentrations of PSCP (Figure 6D, panels 0.2X-HED-PSCP and 1X-HED-PSCP). These observations suggest that PSCP can prevent inflammation induced caudal fin degeneration, suggesting the osteo-protective effects of its components.

### 3.7 Patanjali Special Chyawanprash Reduced Pro-Inflammatory Cytokine Release *in vitro* by Moderating NF-κB Signaling at a Cytological Safe Concentration

**FIGURE 7:**
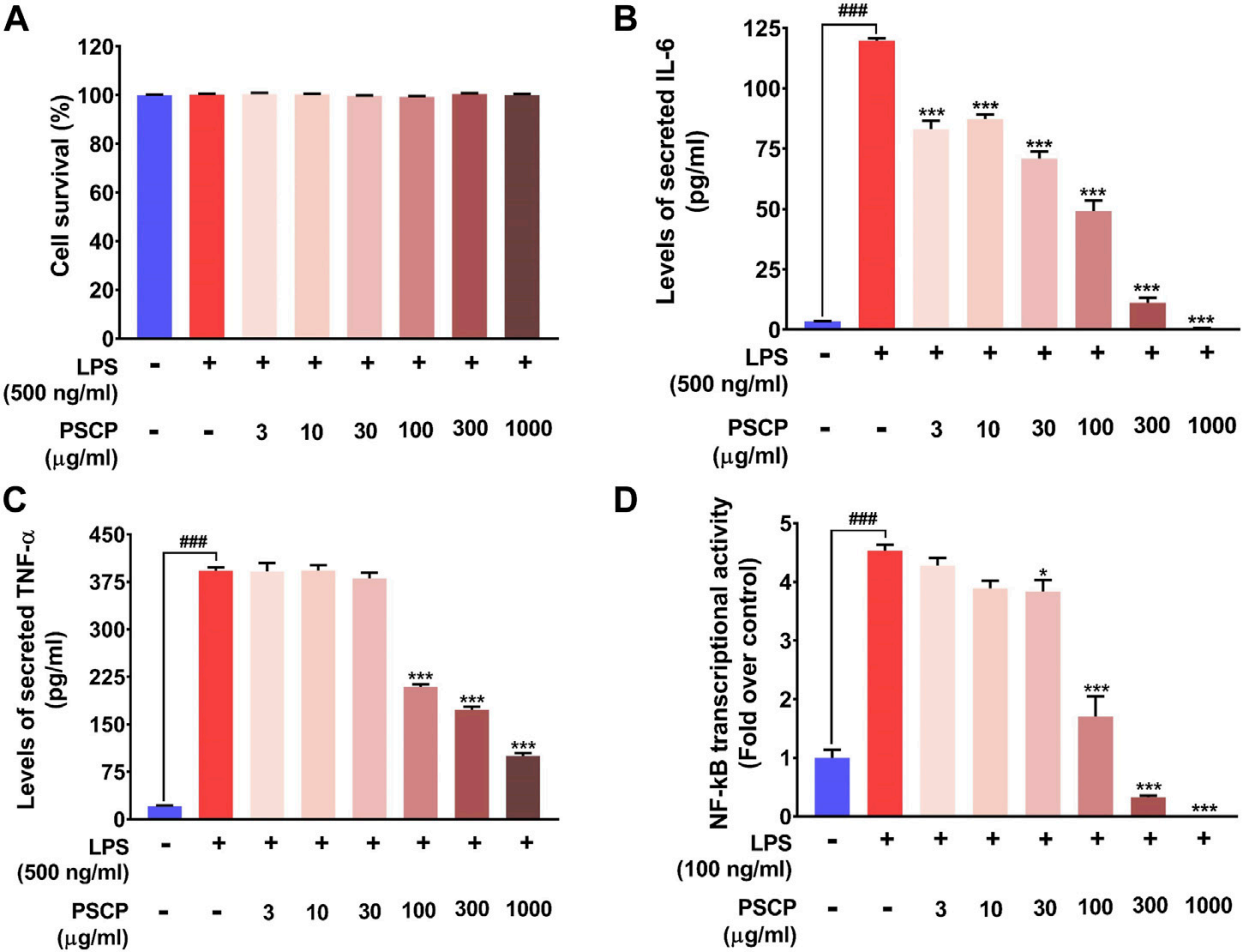
PSCP inhibited pro-inflammatory cytokine release *in vitro* by modulating NF-κB signaling at cyto-safe doses. **(A)** Cytotoxicity analysis of different concentrations of PSCP used to treat LPS-induced THP-1 cells demonstrating the suitability of the *in vitro* inflammatory model for subsequent experiments. **(B,C)** Quantitative representations of the effects of PSCP treatment on the levels of IL-6 **(B)** and TNF-α **(C)** secreted in the cell supernatant by differentiated THP-1 cells in response to LPS-induced inflammation. **(D)** Effect of PSCP on LPS-induced NF-κB signaling measured through SEAP reporter assay. Data is represented as mean ± SEM. Statistical significance was analyzed through one-way ANOVA and represented as ### for *p* < 0.001, when significantly different from the normal cells, untreated with PSCP and LPS, or as * and *** for *p* < 0.05 and <0.001, respectively, when the comparison was with the PSCP-untreated LPS-treated group.

From *in vivo* observations, PSCP loaded fish feeds could reduce the transcript levels of IL-6, IL-1β, and TNF-α significantly. In order to verify this observation *in vitro*, the effect of PSCP on cytokine release was evaluated using differentiated, LPS-induced inflammatory THP-1 cells. But prior to that, the safety of treating these cells with PSCP herbal extracts was established through alamar blue staining, a method used to ascertain cell survival (Figure 7A). PSCP herbal extract even at 1,000 μg/ml was found to be safe on THP-1 cells for a straight 30 h. Cytokine release was therefore assayed using the cytotoxicity assay verified concentrations of PSCP. The 24 h PSCP pre-treated cells were co-treated with LPS for another 6 h. Subsequent estimation of pro-inflammatory cytokines levels in the supernatant of PSCP-untreated, LPS-induced cells, as expected, showed greater than 100- and 350-fold increases in IL-6 and TNF-α, respectively, compared to their normal counterparts (Figures 7B,C). PSCP treatment exhibited significant dose–dependent reductions in IL-6 release by the LPS-induced THP-1 cells, starting with its lowest studied concentration of 3 μg/ml **(** Figure 7B). In the case of TNF-α, the effective reduction and dose-dependency were noted with 100 μg/ml and higher concentrations of PSCP studied (Figure 7C). Additionally, in order to have a mechanistic perception of the anti-inflammatory property of PSCP, we conducted *in vitro* NF-κB-driven SEAP reporter assay. LPS-induced inflammatory responses manifest through NF-κB signaling (Liu et al., 2017). Based on this premise, expressions of SEAP from an NF-κB regulatable promoter, in THP1-Blue™ NF-κB cells were used to decipher the effect of PSCP on NF-κB signaling. In the absence of PSCP pre-treatment, the cells expressed nearly 4.5-fold more SEAP upon LPS induction, when compared to the normal ones. SEAP levels gradually decreased with PSCP treatments at increasing amounts, the reduction being statistically discernible with a marked dose-dependency from 30 μg/ml onward (Figure 7D). Altogether, these *in vitro* observations corroborated the *in vivo* outcomes that PSCP is biologically safe and inhibits pro-inflammatory cytokine releases. From the outcomes of SEAP reporter assay, this anti-inflammatory effect of PSCP was attributable to its NF-κB-signaling-modulatory ability.

### 3.8 Compositional Analysis of Patanjali Special Chyawanprash

**FIGURE 8:**
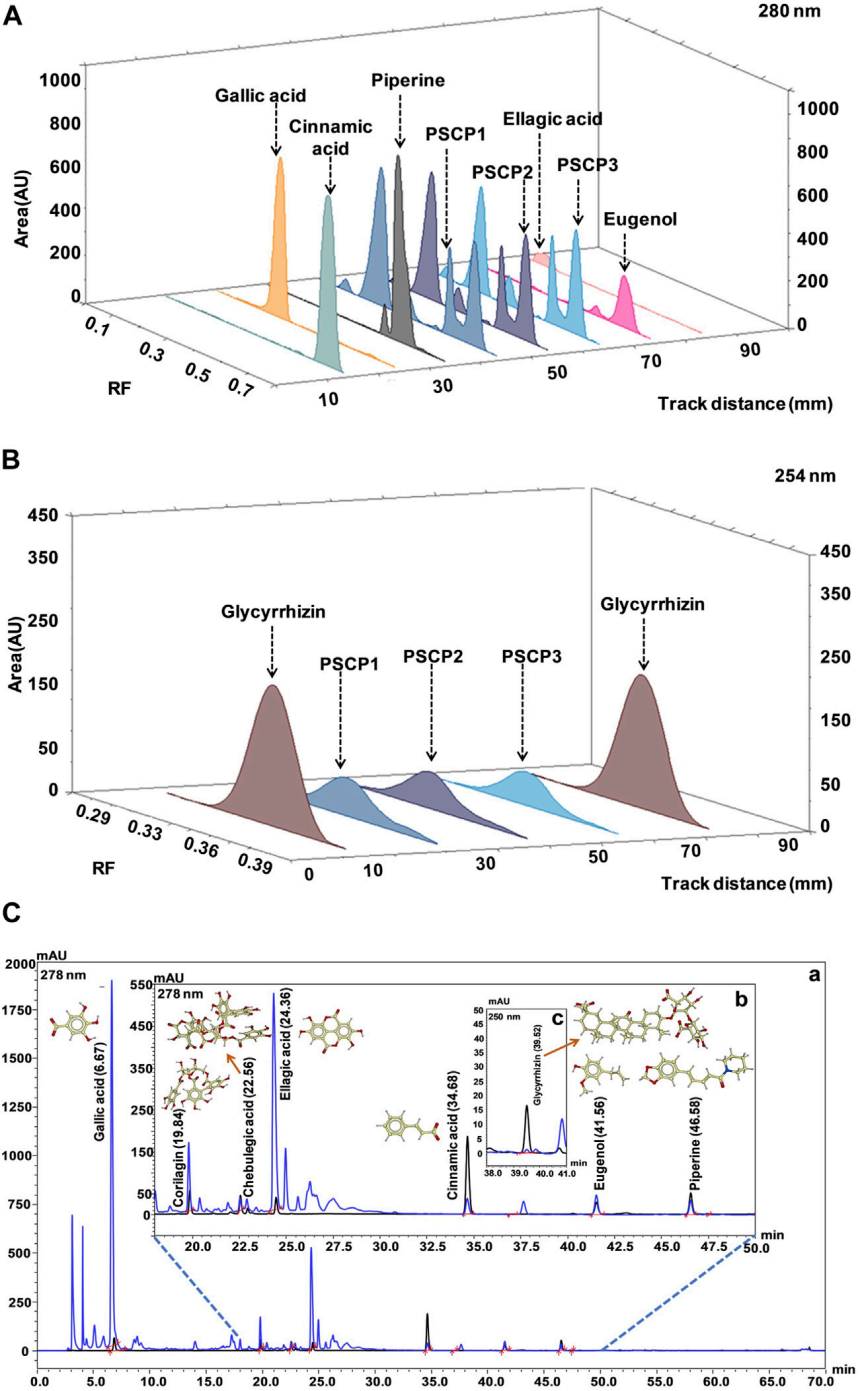
Chemical composition of PSCP. **(A,B)** HPTLC fingerprinting analysis of PSCP shown as chromatograms with the markers Cinnamic acid, Gallic acid, Piperine, Eugenol, and Ellagic acid scanned at 280 nm **(A)** and with Glycyrrhizin as marker scanned at 254 nm **(B)**. **(C)** Overlap chromatograms obtained from HPLC analysis of PSCP (in blue) with standard mix (in black) of Gallic acid (6.67 min) **(a)**, Corilagin (19.84 min), Chebulagic acid (22.56 min), Ellagic acid (24.36 min), Cinnamic acid (34.68 min), Eugenol (41.56 min), and Piperine (46.58 min) **(b)** at 278 nm, and Glycyrrhizin (39.52 min) **(c)** at 250 nm.

As observed above, PSCP effectively ameliorated LPS induced pathologies in zebrafish which suggested strong immunity boosting. Therefore, the composition of PSCP was subjected to chemical analysis through HPTLC and HPLC in order to understand the source of this effect. Chemical fingerprinting through HPTLC revealed that Cinnamic acid, Gallic acid, Piperine, Eugenol, Ellagic acid, and Glycyrrhizin were present in PSCP (Figures 8A,B). HPLC analysis confirmed the presence of these phytochemicals in PSCP, in addition to Corilagin and Chebulagic acid (Figure 8C). Both HPTLC and HPLC analyses revealed that Gallic acid was the major phytochemical present in PSCP (5.02 ± 1.10 and 5.42 ± 1.19 μg/mg according to HPTLC and HPLC, respectively) followed by Ellagic acid, Eugenol, Corilagin, Piperine, Chebulagic acid, Glycyrrhizin, and Cinnamic acid in that order. HPTLC and HPLC analyses revealed a comparable compositional profile of PSCP. Batch to batch compositional inconsistency is a major current issue with different brands of CPs (Sharma et al., 2019). Here, we have analyzed the composition of three independent batches of PSCP and observed that this variation is minimal in our case (Table 2). The compositional analysis revealed that PSCP is enriched with phytocompounds which are potent anti-inflammatory and antioxidant agents.

**TABLE 2:** Phytochemical composition of PSCP.

Name of the phytochemical	Amount determined through HPLC (µg/mg)			Average amount determined by HPLC (µg/mg) (mean ± SEM)	Amount determined through HPTLC (µg/mg)			Average amount determined by HPTLC (µg/mg) (mean ± SEM)
Batch nos. Of PSCP	ADA2000017	ADA1900025	ADA2000028		ADA2000017	ADA1900025	ADA2000028	
Gallic acid	7.18	5.92	3.16	5.42 ± 1.19	6.81	5.24	3.01	5.02 ± 1.10
Ellagic acid	2.57	2.21	2.22	2.33 ± 0.12	2.46	2.43	2.31	2.4 ± 0.05
Eugenol	0.5	0.64	0.51	0.55 ± 0.05	0.52	0.67	0.49	0.56 ± 0.06
Corilagin	0.39	0.43	0.6	0.47 ± 0.06	—	—	—	—
Piperine	0.28	0.16	0.29	0.24 ± 0.04	0.21	0.13	0.22	0.2 ± 0.03
Chebulagic acid	0.21	0.12	0.1	0.14 ± 0.03	—	—	—	—
Glycyrrhizin	0.05	0.06	0.06	0.06 ± 0.003	0.05	0.06	0.06	0.06 ± 0.003
Cinnamic acid	0.06	0.05	0.02	0.04 ± 0.01	0.04	0.03	0.02	0.03 ± 0.01

## 4 Discussion

**FIGURE 9:**
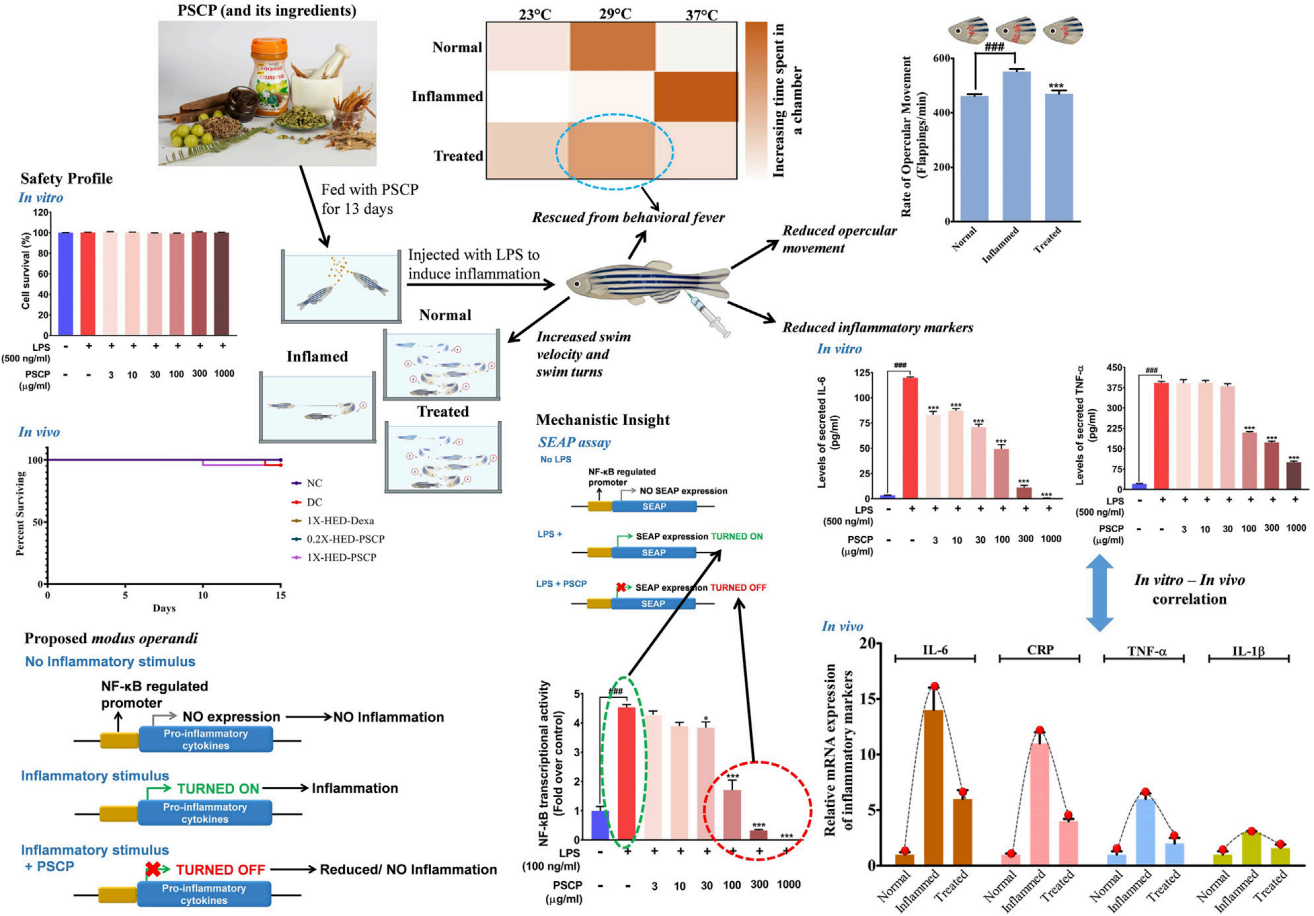
Proposed model for immunomodulatory activity of PSCP. PSCP, which is made from different herbs, is capable of preventing inflammatory responses in zebrafish and, consequently, averts several concomitant phenotypic manifestations, like behavioral fever, hyperventilation, and reduced physical activeness. The *in vitro* observations corroborated with those obtained from the *in vivo* study. SEAP reporter assay helped in identifying that PSCP exerts its anti-inflammatory effects by targeting NF-κB transcriptional activity.

Chyawanprash is a popular Indian classical health supplement known for its particular demands during winters. It is recommended for all age groups for improving the body’s holistic capacity to combat against weather and environmental changes (Sharma et al., 2019). In fact, based on the classical texts of Ayurveda (the system of traditional Indian medicine), Ministry of Health and Family Welfare, Government of India, endorsed the consumption of Chyawanprash in its advisory for immunity boosting during the COVID-19 pandemic (Ministry of AYUSH, Government of India, 2020) and later on for post-COVID-19 management (Ministry of Health and Family Welfare, Government of India, 2020). While the phytochemical constitution of Chyawanprash makes its anticipated immunity boosting property obvious, its anti-inflammatory effect still required attention, particularly when it is being recommended as a health supplement in the post-COVID-19 times. Therefore, besides monitoring batch-to-batch consistency, we evaluated the anti-inflammatory property of PSCP, using the LPS induced inflammation model of zebrafish. LPS injection triggers several inflammatory pathologies in zebrafish, such as behavioral fever, reduced activeness and locomotory agility, hyperventilation, cardiac dysfunction manifested as blood clots in the form of skin hemorrhage, and osteo-degeneration as visible loss of a caudal fin. PSCP could ameliorate all these pathological manifestations of LPS induced inflammation in zebrafish at a human equivalent dose. We also observed that PSCP prevented pro-inflammatory cytokine response triggered by inflammation, thereby exhibiting clear implications on adaptive immune response. SEAP reporter assay provided an insight that PSCP affects NF-κB transcriptional activity. The observed reduction in the levels of secreted IL-6 and TNF-α can be traced back to this reduced transcriptional activity of NF-κB due to PSCP treatment. After all, NF-κB is a master transcriptional regulator when it comes to raising an inflammatory response (Liu et al., 2017). All in all, the outcomes of this study convincingly established the anti-inflammatory ability of PSCP with an implication of NF-κB signaling to be the mechanistic target (Figure 9).

Compositional analyses of different batches of PSCP confirmed Gallic acid to be the major phytochemical present in it, followed by Ellagic acid. Eugenol, Corilagin, Piperine, Chebulegic acid, Glycyrrhizin, and Cinnamic acid are present in that order. Inflammation and oxidative stress are closely related pathophysiological events, one reinforcing the other (Biswas, 2016). The polyphenol, Gallic acid is known to efficiently strike a balance between pro- and anti-inflammatory processes, besides being an excellent scavenger for reactive oxygen species and, thus, a potent antioxidant (Badhani et al., 2015). Likewise, Ellagic acid is also known for its anti-inflammatory effect and antioxidant potential (Han et al., 2006; Corbett et al., 2010; BenSaad et al., 2017). Among other phyto-constituents of PSCP, Eugenol had been reported to have an ameliorative effect on LPS induced lung inflammation in mice through moderating redox status (Magalhães et al., 2010; Huang et al., 2015). An anti-inflammatory effect of Piperine reported in IL-1β stimulated synoviocytes from patients with rheumatoid arthritis and carrageenan and collagen induced arthritic rat models are also attributed to its antioxidant potential (Bang et al., 2009; Umar et al., 2013). Likewise, Corilagin, Chebulagic acid, Glycyrrhizin, and Cinnamic acid owe their anti-inflammatory effects to their antioxidant potentials (Kinoshita et al., 2007; Li et al., 2011; Liao et al., 2012; Sasidharan et al., 2012; Sova, 2012).

LPS induced inflammation in zebrafish recapitulated bacterial sepsis in humans characterized by cardiac arrhythmia, inflammation, and disseminated intravascular coagulation (Semeraro et al., 2010; Philip et al., 2017; Shahreyar et al., 2018). Several inflammatory diseases are associated with dysfunctional bone remodeling (Lacativa and Farias, 2010; Li et al., 2019). Loss of caudal fin in zebrafish following LPS induced inflammation might be a recapitulation of the loss of bone mineral density as reported in patients with sepsis (Philip et al., 2017; Hongo et al., 2019), particularly when microscopic anatomy of zebrafish fin is known to resemble that of human bones (Bergen et al., 2019). This suggests that septic arthritis can be modelled onto zebrafish. Moreover, LPS was shown to exacerbate collagen induced rheumatoid arthritis in mice (Tanaka et al., 2013). Piperine, one of the constituting phytochemicals of PSCP, is effective against collagen induced rheumatoid arthritis in rats (Umar et al., 2013). Given this, the most intriguing observation was the osteoprotective effect of PSCP evident from no loss of caudal fin when zebrafish were fed with PSCP infused pellets. Our zebrafish model requires validation for one or the other of septic and rheumatoid arthritis. Nevertheless, the observed osteoprotective role of PSCP, under the given conditions, suggests its nutraceutical potential against these diseases, and warrants further research in this direction. Such possibilities require extensive explorations building scope for a whole new study.

## 5 Conclusion

Taken together, we report that Patanjali Special Chyawanprash (PSCP) could efficiently prevent the inflammatory manifestations of LPS *in vitro* in THP-1 macrophages and *in vivo*, in a zebrafish model of inflammation. Additional *in vitro* reporter assay indicated that NF-κB signaling is being targeted by PSCP for anti-inflammatory manifestations. On a final note, it could be concluded that this study has presented the *in vivo* validation of the anti-inflammatory property and *in vitro* insight into the mode-of-action of the traditional medicinal food, Chyawanprash.

## Data Availability

The original contributions presented in the study are included in the article/Supplementary Material, further inquiries can be directed to the corresponding author.
